# Single and Competitive Adsorption of 17α-Ethinylestradiol and Bisphenol A with Estrone, β-Estradiol, and Estriol onto Sediment

**DOI:** 10.3390/md12031349

**Published:** 2014-03-07

**Authors:** Yu Li, Chen Zhang, Shanshan Li, Changzhi Zhou, Xiaopeng Li

**Affiliations:** 1 Resources and Environmental Research Academy, MOE Key Laboratory of Regional Energy Systems Optimization, North China Electric Power University, No. 2, Beinong Road, Changping District, Beijing 102206, China; E-Mails: 1112102045@ncepu.edu.cn (Z.C.); zhouchangzhi@ncepu.edu.cn (Z.C.); 1112200025@ncepu.edu.cn (L.X.); 2 Department of Civil and Environmental Engineering, University of Louisville, Louisville, KY 40292, USA; E-Mail: s0li0013@louisville.edu

**Keywords:** bisphenol A, 17α-ethinylestradiol, competitive adsorption, sediment, selectivity

## Abstract

The competitive adsorption of bisphenol A (BPA) and17α-ethinylestradiol (EE2) with different endocrine disrupting compounds (EDCs), such as estrone (E1), β-estradiol (E2), and estriol (E3) was investigated in the water-sediment system. The primary and interaction effects of coexisted EDCs on the adsorption of BPA and EE2 were studied in binary and multiple systems. The adsorption selectivity of sediment at different initial concentrations of EDCs was also considered, based on the distribution coefficient (β). In binary systems, coexisted EDCs exhibited a positive effect on the adsorption of BPA, while E3 showed a negative effect on the adsorption of EE2. In ternary systems, the interaction of E1*E3 and E2*BPA showed a synergistic effect on the sorption of BPA and EE2, respectively. In quaternary systems, the interaction of E1*E2*E3 showed a synergistic effect on the adsorption of both BPA and EE2. In the quinary system, coexisted EDCs all showed an antagonistic effect on the adsorption of BPA and EE2, which indicated that the coexisted EDCs competed for adsorption with BPA and EE2. EDCs in the E2-EE2-BPA system presented a superior selectivity of sediment with β values of 43.48–87.86. The order of sediment selectivity (E1 > EE2 > E2 > E3 > BPA) in binary systems was in agreement with EDCs’ adsorption capacity, which suggested that the adsorption was dominated by partition adsorption.

## 1. Introduction

EDCs are compounds that disrupt wildlife’s reproductive system by imitating and blocking the activity of natural hormones [1]. The synthetic EDCs 17α-ethinylestradiol (EE2) and bisphenol A (BPA) are frequently found in wastewater, marine water, and sediments [2,3]. EE2 is generally used in contraceptive pills [4]. Because of the long half-life, the toxicity of EE2 in fish was determined to be 10–50 times greater than estrone (E1) and β-estradiol (E2) [5]. EE2 usually finds its way into the surface water via the wastewater. After entering the water, EE2 is absorbed by sediments until equilibrium is achieved and, then, the excess EE2 is again released into the water. Previous investigations observed high concentrations of EDCs in sediments and to a lesser extent in air and drinking water [6]. BPA is mainly used as a synthetic plasticiser in the chemical industry and exhibited estrogenic active even at micrograms per litre concentrations [7]. E1, E2, and E3 are mainly released by excreta of human and livestock, and were able to reverse the gender of aquatic animals even at low concentrations (µg/L) [8,9]. Due to the disorderly discharged sewage and residues from conventional wastewater treatment plants, the EDCs are transferred into the sediment-water system [10]. Furthermore, manure spreading worsens EDC transfer into the sediment-water system [11]. Despite the fact that most of the EDCs in wastewater and manure can be removed via various treatment processes, the remaining concentration still is larger than the maximal theoretical safe concentration.

Increasingly, studies have been conducted that focussed on the behaviour of EDCs in sediment/water [12,13,14]. To better understand the adsorption in sediment, the distribution and accumulation of EDCs were studied in sediment-water by researchers [15]. Recently, Sun *et al.* [16] studied the adsorption of EDCs onto different size fractions of sediment. Card and co-workers evaluated and predicted the adsorption behaviour of hormones in sediment [17]. The adsorption of four different EDCs was investigated in a sediment-water system, and the environmental fate of EDCs was studied by Li *et al.* [18]. Although the aforementioned studies examined the distribution and adsorption of EDCs in a sediment-water system, competitive adsorption of multiple EDCs in such a system and the selectivity of the sediment were not explored in detail to date.

In the current study, EE2 and BPA were set as the research targets; and the natural EDCs (E1, E2, and E3) were set as the interferents. The objectives of this work were to: (1) determine the primary and interaction effect of coexisting EDCs on EE2 and BPA in binary and multiple competitive adsorption systems; (2) to estimate the adsorption capacity of EDCs in binary systems; and (3) to estimate the selectivity of the sediment for binary and multiple EDCs in the adsorption process. The study aims to provide a theoretical foundation for the adsorption of multiple EDCs in a water-sediment system.

## 2. Results and Discussion

### 2.1. Differences in Adsorption Capacity of EE2 and BPA in the Binary System

In the binary-EDCs system, the competitive effects of coexisting EDCs on EE2 and BPA are represented by the included angle (tan θ) of the linear isotherms. When tan θ > 0, the adsorption is regarded as promoted by the coexisting EDCs; when tan θ = 0, there is no competitive effect by the coexisting EDCs; when tan θ < 0, the adsorption is restrained by the coexisting EDCs. The correlation coefficients of the isotherms were all greater than 0.90, which indicated that the equations were all well fitted.

The specific variations of tan θ in both single and binary adsorption systems are shown in Table 1. For BPA, the values for tan θ in the binary-EDCs system were all larger than 0, confirming that the coexisting EDCs promote the adsorption of BPA. The adsorption capacity of BPA reached an optimum value in the condition of coexisting with E1. For EE2, the value of tan θ is less than 0 when coexisting with E1, E2, and BPA. This result indicated that the presence of E1, E2, and BPA results in a restraining effect on the adsorption of EE2, and E1, E2, and BPA might be competing with EE2 for adsorption binding. The value for tan θ was larger than 0 for the condition that EE2 coexisted with E3, which suggests that E3 promoted the adsorption of EE2 and the adsorption reached the optimum value.

**Table 1:** The effect of coexisting EDCs on EE2 and BPAs in binary adsorption.

**EDCs**	**Parameters**	**Conditions**				
		**Single**	**Coexisted with E1**	**Coexisted with E2**	**Coexisted with EE2**	**Coexisted with E3**
BPA	*R*^2^	0.9931	0.9689	0.9658	0.9619	0.9424
	*k*	0.0072	0.0128	0.0112	0.0083	0.0078
	tan θ_i_	-	0.0057	0.0040	0.0011	0.0006
**EDCs**	**Parameters**	**Conditions**				
		**Single**	**Coexisted with E1 **	**Coexisted with E2**	**Coexisted with E3**	**Coexisted with BPA**
EE2	*R*^2^	0.9192	0.9687	0.9699	0.9210	0.9340
	*k*	0.03223	0.0254	0.02705	0.03381	0.03149
	tan θ_i_	-	−0.0068	−0.0052	0.0016	−0.0007

**Figure 1:**
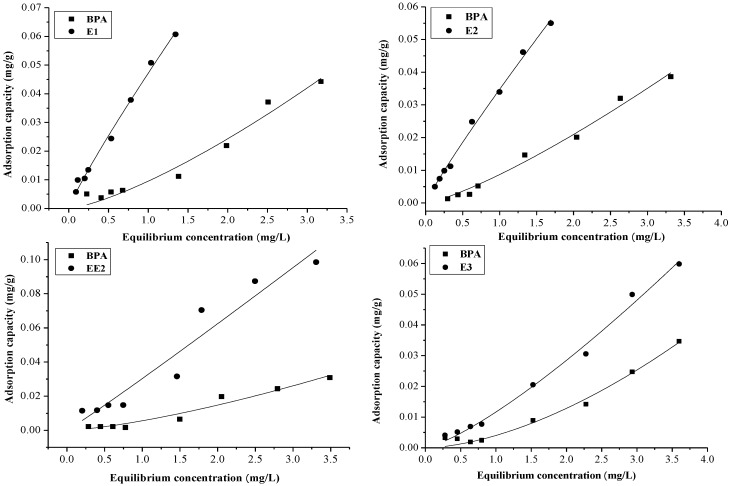
The adsorption capacity of EDCs in the BPA binary system.

**Figure 2:**
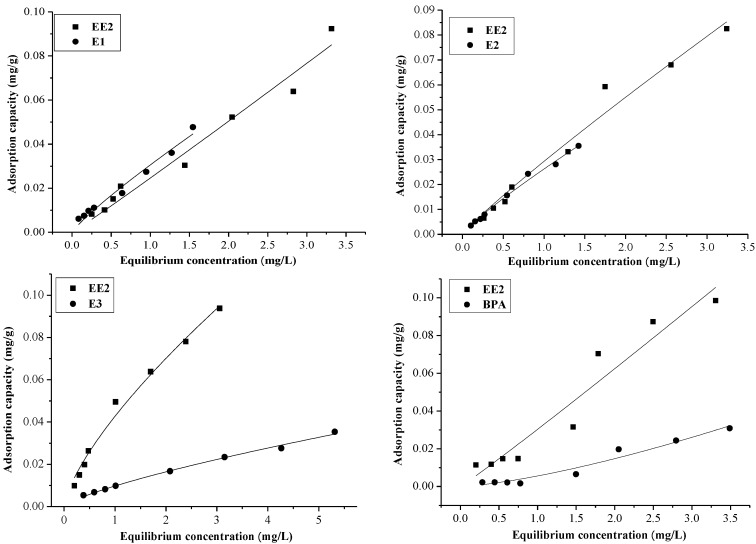
The adsorption capacity of EDCs in the EE2 binary system.

Furthermore, the adsorption capacities of EE2 and BPA in single and binary systems were investigated, and the thermodynamics of EDCs adsorption were fitted according to the Fredilich isotherm (Figure 1 and Figure 2). From the Figure 1, it can be seen that the adsorption capacities of EDCs in the BPA binary system were: BPA < E1, BPA < E2, BPA < EE2, and BPA < E3. For the EE2 binary system, Figure 2 shows that the order of the adsorption capacities of the EDCs in the binary system were: EE2 < E1, EE2 > E2, EE2 > E3, and EE2 > BPA. To explore the order of the adsorption capacities of the EDCs in sediment, the adsorption of E2 and E3 in single and binary system were determined, and the order of the adsorption capacities were: E2 > E3. Liu *et al.* [19] previously regarded the adsorption of EDCs on activated carbon as a physical process in the study of removal methods. In the present study, we equally assumed that the adsorption of EDCs onto sediment was a physical process and, therefore, the order of adsorption capacities of the EDCs in the binary system would be: E1 > EE2 > E2 > E3 > BPA.

### 2.2. The Differences of Adsorption Capacity of Multiple EDCs

In multiple-EDCs systems, the competitive effects between EE2 (or BPA) and natural EDCs were equally represented by the included angle (tan θ) of the isotherms. The difference is: the interactions of coexisting EDCs were the focus of this research. When tan θ > 0, the adsorption is regarded as promoting synergism between coexisting EDCs; when tan θ = 0, there is no interaction effect between the coexisting EDCs; when tan θ < 0, the adsorption is reduced by the antagonistic effect between coexisting EDCs.

#### 2.2.1. The Adsorption of BPA and EE2 in a Ternary EDCs System

The values of tan θ are shown in Table 2. The results in Table 2 show that, for BPA, tan θ of the ternary EDCs system are all positive, which suggests that the coexisting EDCs synergistically affected the adsorption of BPA. According to the tan θ values, this synergistic effect of coexisting EDCs could be induced according to the following sequence: E1*E3 > E2*E3 > E1*E2 and E1*EE2 > E2*EE2 > EE2*E3. For EE2, the synergistic interaction of coexisting EDCs were in the following order: E2*BPA > E1*E3 > E2*E3 > E1*BPA. The antagonistic effect of coexisting EDCs were as follows: E1*E2 > E3*BPA, which confirms that E1*E2 and E3*BPA restrained the adsorption of EE2 and the extent of restraining was: E1*E2 > E3*BPA.

**Table 2:** The variation of adsorption capacity of BPA and EE2 in a ternary EDCs system.

**EDCs**	**Parameters**	**Conditions**			
		**Single**	**Coexisted with E1 and E2**	**Coexisted with E2 and E3**	**Coexisted with E1 and E3**
**BPA**	*R*^2^	0.9955	0.9868	0.9876	0.9818
	*k*	0.0072	0.0109	0.0114	0.0122
	tan θ_i_	-	0.0037	0.0042	0.0050
	**Parameters**	**Conditions**			
		**Single**	**Coexisted with E2 and EE2**	**Coexisted with E1 and EE2**	**Coexisted with EE2 and E3**
	*R*^2^	0.9955	0.9909	0.9349	0.9947
	*k*	0.0072	0.0105	0.0109	0.0094
	tan θ_i_	-	0.0033	0.0037	0.0022
**EDCs**	**Parameters**	**Conditions**			
		**Single**	**Coexisted with E1 and E2**	**Coexisted with E1 and E3**	**Coexisted with E2 and E3**
**EE2**	*R*^2^	0.9236	0.9641	0.9922	0.9856
	*k*	0.0322	0.0241	0.0408	0.0373
	tan θ_i_	-	−0.0081	0.0086	0.0051
	**Parameters**	**Conditions**			
		**Single**	**Coexisted with E1 and BPA**	**Coexisted with E2 and BPA**	**Coexisted with E3 and BPA**
	*R*^2^	0.9236	0.9514	0.9969	0.9884
	*k*	0.0322	0.0352	0.9029	0.0307
	tan θ_i_	-	0.0030	0.8461	−0.0015

#### 2.2.2. The Adsorption of BPA and EE2 in a Quaternary EDCs System

To better understand the effect of interaction of coexisting EDCs, the adsorption of BPA and EE2 in a quaternary system were studied (Table 3).

**Table 3:** The variation of adsorption capacity of BPA and EE2 in a quaternary EDCs system.

**EDCs**	**Parameters**	**Conditions**				
		**Single**	**With E1, E2, and EE2**	**With E1, E2, and E3**	**With E1, EE2, and E3**	**With E2, EE2, and E3**
BPA	*R*^2^	0.9955	0.9931	0.9952	0.9693	0.9899
	*k*	0.00715	0.0098	0.0396	0.0058	0.0130
	tan θ_i_	-	0.0026	0.0324	−0.0013	0.0058
**EDCs**	**Parameters**	**Conditions**				
		**Single**	**With E1, E2, and E3**	**With E1, E2, and BPA**	**With E1, E3, and BPA**	**With E2, E3, and BPA**
EE2	*R*^2^	0.9236	0.9794	0.9899	0.9965	0.9329
	*k*	0.0322	0.0396	0.0333	0.0290	0.0255
	tan θ_i_	-	0.0074	0.0011	−0.0032	−0.0067

For the adsorption of BPA and EE2 in the quaternary EDCs system, the influence of ternary interactions by coexisting EDCs was studied. For the adsorption of BPA, the presence of E1*E2*EE2, E1*E2*E3, and E2*EE2*E3 showed synergistic effects, and these ranked according to: E1*E2*E3 > E2*EE2*E3 > E1*E2*EE2. However, the interaction of E1*EE2*E3 showed an antagonistic effect on the adsorption of BPA. For the adsorption of EE2, the presence of E1*E2*E3 and E1*E2*BPA showed a synergistic effect, which was E1*E2*E3 > E1*E2*BPA. Conversely, E1*E3*BPA and E2*E3*BPA showed an antagonistic effect on the adsorption of EE2, and the order was: E2*E3*BPA > E1*E3*BPA.

#### 2.2.3. The Adsorption of BPA and EE2 in a Quinary EDCs System

As it was shown in Table 4, for the adsorption of BPA and EE2, tan θ < 0 demonstrated that competitive adsorption occured in the presence of E1, E2, EE2, E3, and E1, E2, E3, BPA, respectively. In the quinary EDCs system, interaction of E1*E2*EE2*E3 showed an antagonistic effect on the adsorption of BPA and E1*E2*E3*BPA antagonistically affected the adsorption of EE2.

**Table 4:** The variation of adsorption capacity of BPA and EE2 in a quinary EDCs system.

**EDCs**	**BPA**		
Parameters	*R*^2^	*k*	**tan θ_i_**
Single	0.9955	0.0072	-
With E1, E2, EE2, and E3	0.9961	0.0030	−0.0042 (Antagonistic effect)
**EDCs**	**EE2**		
**Parameters**	*R*^2^	*k*	**tan θ_i_**
Single	0.9236	0.0322	-
With E1, E2, E3, and BPA	0.9848	0.0212	−0.0110 (Antagonistic effect)

### 2.3. Selectivity of Sediment for EDCs

#### 2.3.1. Selectivity of Sediment for EDCs in a Binary System

**Figure 3:**
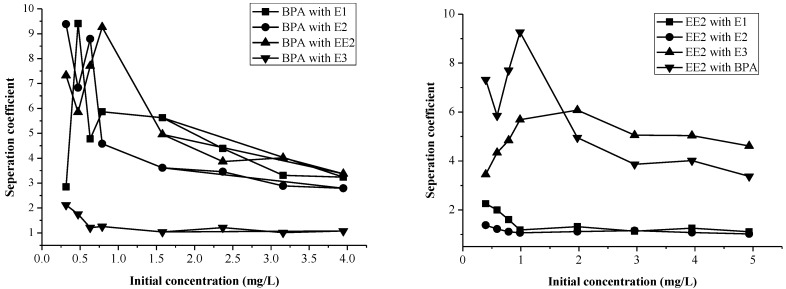
The separation coefficients (β_a/b_) of BPA and EE2 in a binary system.

The adsorption of pollutions not only depends on the adsorption capacity and coexisting contaminants, but also on the selectivity of the sediment. The separation coefficients (β_a/b_) of the EDCs in binary systems were studied at initial concentrations of 1.0–5.0 mg/L (Figure 3).

In the binary system of BPA, the β_a/b_ of the EDCs was in the order of: β_E1/BPA_ > β_EE2/BPA_ > β_E2/BPA_ > β_E3/BPA_. In the binary system of EE2-BPA, β_EE2/BPA_ was larger than β_E1/EE2_, β_EE2/E2_, and β_EE2/E3_ when the initial concentration was in the range of 0–1 mg/L. When the initial concentration was 1–5 mg/L, β_EE2/BPA_ was larger than β_EE2/E3_ and the value of β_E1/EE2_ was close to β_EE2/E2_. Figure 3 infers that the initial concentration of EE2 has a major impact on the selectivity of the sediment. The order of sediment selectivity of EDCs is accordance with the adsorption capacity, which was E1 > EE2 > E2 > E3 > BPA. These results show that the higher the selectivity of the sediment, the stronger the adsorption capacity of the EDCs. Furthermore, the results indicate that the adsorption of EDCs onto surface sediment is dominated by partition adsorption.

#### 2.3.2. The Selectivity of Sediment for EDCs in a Ternary System

**Figure 4:**
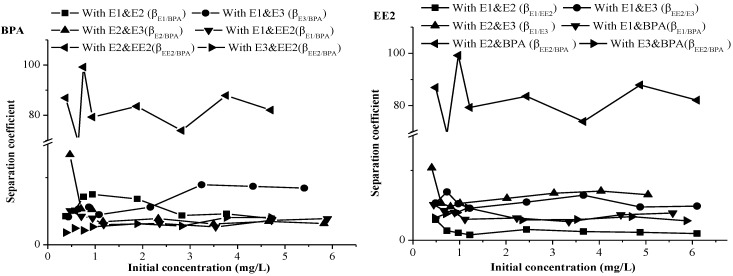
The separation coefficient (β_a/b_) of BPA and EE2 in a ternary adsorption system.

A series of ternary EDCs systems were chosen to analyze the sediment selectivity of adsorption and the adsorption selectivity was measured via β_a/b_ (Figure 4).

Figure 4 shows that β_E1/BPA_ was in the range of 43.48–87.86 in the E2-EE2-BPA system, which suggest that the selectivity of the sediment for EDCs in the E2-EE2-BPA system was superior to other ternary systems. For the adsorption of BPA, the selectivity of sediment was minimal in the BPA-E3-EE2 system and is in agreement with the results for E3*EE2, which has a weakly promoting effect on the adsorption. For the adsorption of EE2, the selectivity of the sediment showed a minimum value in the EE2-E1-E2 system. This can be attributed to the antagonistic effect of E1 *E2 in the system.

#### 2.3.3. The Selectivity of Sediment for EDCs in a Quaternary and Quinary System

**Figure 5:**
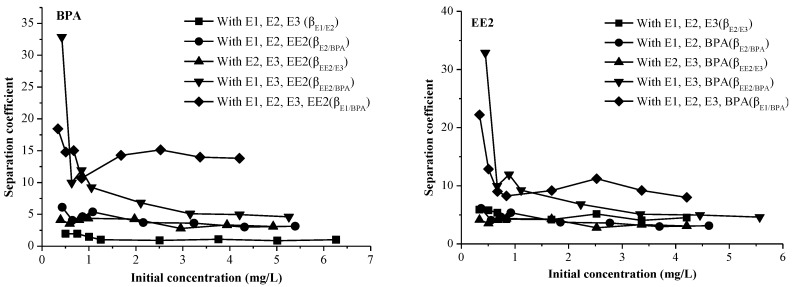
The separation coefficient (β_a/b_) of BPA and EE2 in a quaternary and quinary adsorption system.

To simulate the behaviour of the EDCs in natural water, the adsorption selectivity of BPA and EE2 in both quaternary and quinary system were studied at different initial concentrations (Figure 5). At the initial concentration of 0–1 mg/L, the adsorption selectivity of BPA and EE2 in the E1-E2-EE2-BPA system was obviously superiority and decreased sharply as the initial concentration increased. In the range of 1–5 mg/L, the adsorption selectivity of the EDCs in the quinary system reached its maximum. The selectivity of all EDCs decreased with increasing initial concentration, which suggests that higher concentrations of substrate has a negative effect on the selectivity of the sediment.

## 3. Experimental and Methods

### 3.1. Physicochemical Properties of Sediment

The sediment samples used in this study were directly collected from a depth of 0–5 cm near the Songhua River in Jilin City, China.

### 3.2. Materials and Chemicals

E1, E2, EE2, and E3 (purity > 97%) were purchased from Sigma-Aldrich (Munich, Germany). Methanol and acetonitrile (HPLC grade) were obtained from Burdick & Jackson (Honeywell, Morristown, NJ, USA). NaOH (AR) and HCl (AR) were purchased from the Beijing Chemical Engineering Factory (Beijing, China). CaCl_2_ was purchased from the Xilong Chemical Plant of Shantou, Guangdong. NaN_3_ (AR) was obtained from the Tianjin Fuchen Chemical Reagents Factory. Sediments were collected from the Songhua River in the Jilin Province, China.

A 1200-HPLC (Agilent Company, Santa Clara, CA, USA), equipped with two model pumps (G1312A), an in-line degasser (G1322A), a column oven (G1316A), and a fluorescence detector (G1321A), was used for HPLC analysis. The injection loop volume was 20.0 µL, and a Zorbox SB-C18 column (250 mm × 4.6 mm; 5 µm) was used for the separations. A 2-16K centrifuge (Sigma, Munich, Germany), a FA-1004 analytical balance (Shanghai Hengping Science Instrument Company, Shanghai, China), and Milli-Q ultrapure water (Millipore, Billerica, MA, USA) were also used.

### 3.3. Sorption Isotherms of EDCs

A 5.0 mg/L standard solution of E1, E2, EE2, E3, BPA, and two co-existing EDCs was prepared. Batch experiments were conducted to investigate the adsorption behaviour of the EDCs. For each determination, 0.7 ± 0.0001 g of the surface sediments (SSs) was mixed with 40 mL of the EDCs solution at different concentrations; the concentrations of the EDCs were 0.4, 0.6, 0.8, 1.0, 2.0, 3.0, 4.0, and 5.0 mg/L, respectively. The solutions contained different EDCs and 200 mg/L of NaN3 to minimise biological activity. NaOH and HCl (both 0.1 mol/L) were used to set the pH to 6.70 ± 0.03. The conical flask was placed in a shaker for 24 h at room temperature. Each sample was filtered with a 0.45 µm membrane filter and transferred into 2 mL amber vials for high performance liquid chromatography (HPLC) analysis. Duplicate experiments were conducted to account for experimental error and to investigate the reproducibility of the results. A blank experiment was performed as a control.

### 3.4. Analysis of EDCs

The EDCs were separated and quantified using a gradient elution procedure. The mobile phase was composed of water (A) and methanol (B). The procedure for E1 was 20% A and 80% B; the procedure for E2, EE2, E3, and BPA was 25% A and 75% B. The UV wavelength for compound detection was λ = 280 nm. The elution procedure for the EDCs was all 30% A and 70% B in binary, ternary, and quaternary systems. The elution procedure settings for all the EDCs were as follows: 0–5 min, 70% B; 5–10 min, 70%–85% B; and 10–18 min, 85% B. The flow rate of the mobile phase was 1.0 mL/min. The column temperature was maintained at 30 °C. The excitation wavelength of the fluorescence detector was fixed at 230 nm, and the emission wavelength was set to 315 nm.

### 3.5. Variation of Adsorption Capacity

**Figure 6:**
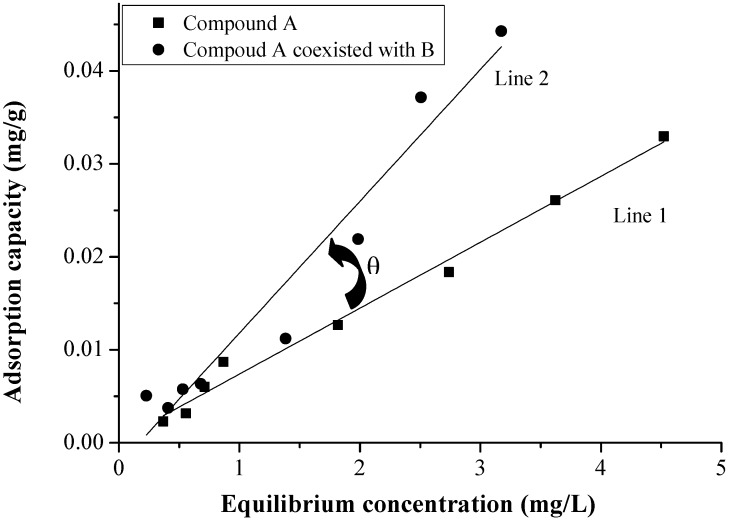
The included angle of isotherms 1 and 2.

The thermodynamics isotherm of EDCs was simulated via the following linear isotherm:

*Q_e_* = *kC_e_* (1)

where *Q_e_* is the concentration of the solid-phase (mg/g), *C_e_* is the concentration of the equilibrium solution phase (mg/L), and k is the Langmuir fitting parameter (L/mg). Figure 6 showed the differences of EDCs adsorption.

Figure 6 describes the isotherms for two conditions: line 1 is the isotherm of compound A in a single adsorption system, and line 2 is the isotherm of compound A coexisting with B in the binary system. There is an acute angle between line 1 and line 2. To quantify the differences between EDCs under a variety of conditions, the acute angle was calculated using the following equation.

tan θ = (*k*_2_ − *k*_1_)/(1 + *k*_1_ × *k*_2_) (2)

where *k*_1_, *k*_2_ are the gradients of line 1 and 2, respectively. Theta (θ) is the angle between line 1 and 2, which indicates the changes in adsorption capacity under two different adsorption conditions. The results show that tan θ was in the range of −1 to 1. The negative value of tan θ shows the competitive effect of coexisting compounds on the target compound, whereas positive values of tan θ show the advanced effect of coexisting compounds on the target compound.

### 3.6. The Selectivity of Sediment for EDCs

*D* = *V*/*m* (*ρ*_0_/*ρ_t_* − 1) (3)

*Β* = *D*_a_/*D*_b_ (4)

where *D* is partition ratio between the sediment and water, *V* is the volume of EDCs solution (L), *ρ*_0_ is the concentration of original solution (mg/L), and *ρ_t_* is the solvent concentration when the adsorption reached equilibrium (mg/L), *m* is the quality of sediment (g). *B* is the partition coefficient between two components.

## 4. Conclusions

The interaction effect of coexisted EDCs on BPA and EE2, and the order of adsorption capacity of BPA and EE2 in binary and multiple adsorption systems were investigated. Additionally, the adsorption selectivity of sediment for EDCs and the influence of different initial concentrations was determined, based on the distribution coefficient (β). The following conclusion can be drawn:

(1) The order of selectivity is accordance with the adsorption capacity (E1 > EE2 > E2 > E3 > BPA) showed the adsorption of EDCs in sediment is dominated in physical adsorption.
(2) The EDCs in ternary and quinary system shows a high selectivity of sediment.
